# Tracking the Effects of Eccentricity on the Integration of Orthographic Information From Multiple Words

**DOI:** 10.5334/joc.446

**Published:** 2025-04-28

**Authors:** Christophe Cauchi, Martijn Meeter

**Affiliations:** 1 ATILF, CNRS & Universitéde Lorraine, France; 2 LEARN! Research Institute. Vrije Universiteit Amsterdam, Netherlands

**Keywords:** reading, perceptual span, orthographic processing, eccentricity, flankers task

## Abstract

In adult readers, the perceptual span is approximately 14–15 characters to the right of the fixated word, corresponding to approximately 5° of visual angle. However, the extent of information processing within this area remains unclear. In the present study, we address this question using a novel adaptation of the flankers task in which the eccentricity of the flankers with respect to the central target word is increased. Fifty-four participants performed a lexical decision task on a central four-letter word flanked by two words of equal length. The flankers were either orthographically related (rock – rock) or unrelated (path – rock) to the target, and their eccentricity varied from 1.65° to 4.29° (center-to-center) in 0.33° steps. Participants’ fixation was controlled by an eye-tracker using the fixation point as a trigger, and stimuli were displayed for 170 ms to avoid any eye movement. Results showed that the effect of unrelated flankers decreased with increasing eccentricity, while there was no effect of eccentricity of related flankers. In particular, the unrelated flankers affected central word processing up to the end of the parafovea. This observation provides evidence that the outer limits of the parafovea are engaged beyond prelexical processing. Lexical frequency influenced the magnitude of both reaction times (RTs) and accuracy rates, but did not interact with any variables. This novel adaptation of the flankers task has potential advantages for investigating the spatial integration of orthographic information across the perceptual span.

## Introduction

Moving window techniques, in which readers only see letters within a predefined window while reading, have shown that the perceptual span of adult readers extends to 14–15 letters to the right of the fixated word (see Rayner, 2009 for a review). With standard experimental settings (3 letters per visual degree), the span is approximately 5° to the right of the fixated word. Although the question of the quantity of textual information contained within the perceptual span has been adequately addressed through moving window reading tasks, the question of how this information is processed within this span may require the use of alternative techniques.

A recent study by Veldre et al. (2023a) tested the ability of readers to process lexical information at different retinal eccentricities of the fixation point. In this work, lexical decision had to be performed on a central target – which could be a word or a pseudoword – and could be presented at five different locations: in the center, 1° left, 2° left, 1° right and 2° right. Distractor flankers were positioned around the target, which could be a different word, a pseudoword or absent. To allow or prevent eye movement in direction of the target, stimulus display times alternated between 100 and 300 ms. The results showed a decline in accuracy concomitant with an increase in RTs with increasing retinal eccentricities of the target location. RTs were higher and accuracy was lower in the presence of distractor flankers than in their absence, and in the presence of word flankers than in the presence of pseudoword flankers.

The work of Veldre et al. is of particular interest for the present study because it supports the idea that the performance of target word recognition is not distributed uniformly across readers’ visual fields. This important work provided a new way for studying the effects of retinal eccentricity within the perceptual span, isolating the phenomenon to be studied – the eccentricity of the information – while avoiding the extra processing imposed by sentence reading, *e.g.*, of syntax and semantics.

In another note, Dare and Shillcock (2013) introduced a lexical version of the Flankers Task paradigm to study how foveal word processing could be affected by flankers in parafovea. In this paradigm, participants performed lexical decision on a central target word or pseudoword that was surrounded by flankers pairs of letters to the left and right separated by a single space. The results showed that flankers directly impacted processing of the central word processing. Grainger et al. (2014) provided a theoretical framework for their interpretation couched in terms of parallel orthographic processing spanning on spatially distinct orthographic stimuli. Subsequently, the flanker effects were replicated with whole words that could be related or unrelated (rock rock rock vs. path rock path). Results showed that flankers related to the target word led to shorter RTs than the unrelated ones (e.g., Snell et al., 2017; Vandendaele & Grainger, 2023; Cauchi et al., 2020). In this work, we propose to use the flankers relatedness to the central word, to track the integration of orthographic information within the perceptual span.

### The present study

In the present study, we propose a novel adaptation of the flankers task, wherein the eccentricity of the flankers is manipulated with respect to the central target word. We start with the assumption that the flankers relatedness effect can be used to probe the spatial integration of orthographic information, by playing with the eccentricity of peripheral words. To achieve this, we instructed participants to respond to a briefly presented flanked central string of letters, indicating whether the string was a word or a pseudoword. Flankers relatedness was manipulated using flankers related (rock rock rock) or unrelated (path rock path) to the target word. Flanker eccentricity was manipulated by placing flankers at different locations from the central target word on an axis varying from 1.65° to 4.29°. We used an eye tracker control with a trigger to ensure that participants fixated on the center of the screen, and the stimulus display time was constrained to 170 ms to limit eye movements during target display. This last point is important given that the high eccentricity of flankers could attract eye movements away from the target with a greater propensity than in flanker experiments with a single space between letter strings. In addition, we manipulated lexical frequency by dividing our stimuli into two sets: the high-frequency set and the low-frequency set.

The main hypothesis of this work is that flankers relatedness effects should decrease with increasing flanker eccentricity. This effect has previously been attributed to facilitation by overlapping input from target and flankers (e.g., Grainger et al., 2014; Meeter et al., 2020), but could also be due to inhibitory influences of lexical competition from unrelated flankers. Our objective was to determine whether these inhibitory influences decrease with eccentricity and to characterize the nature of this decline. An alternative question concerns the foveal load hypothesis (Henderson & Ferreira, 1990). It is not yet clearly established how lexical frequency modulates the word processing in the perceptual span. To provide some answers to this question, we manipulated target frequency, with the idea that a low-frequency target word would present a larger cognitive load than a high-frequency, easily recognized target word. If the foveal load is high with low-frequency words, fewer attentional resources may be available for processing information in the parafovea, leading to reduced relatedness effects and a stronger impact of eccentricity on performance.

## Method

### Participants

A total of 54 university students (45 female) from Vrije Universiteit Amsterdam, ranging in age from 18 to 34 years (M = 21.2, SD = 3.32), gave informed consent to participate in this study. Participants were individually tested in an experimental cubicle in the laboratory. All participants were Dutch native speakers with normal or corrected-to-normal vision and had no history of neurological and/or language impairment. Participants were granted with 10 € or university credits for their participation.

### Apparatus

The experiment was implemented with OpenSesame (Mathôt et al., 2012). Stimuli were displayed in black, lowercase, mono font on a grey background, on an ASUS ROG Strix XG248Q monitor (23.8-inches, 1,920 × 1080 px, 240 Hz). The participant’s eye position was tracked with an EyeLink 1000 eye tracker manufactured by SR Research (Mississauga, ON, Canada). The eye movement cameras are mounted on a headband (one camera for each eye), but the recording was monocular. Participant head position was stabilized on a chin-rest. At 70 centimeters, with a 26-pts font size, the characters subtended .33° of visual angle on the retina.

### Material and Design

One-hundred-and-eighty high-frequency words (mean Zipf = 4.57, SD = .62), and 180 low-frequency words (mean Zipf = 3.10, SD = .36) were used in this experiment. All the words were four-letter Dutch words selected from the SUBTLEX database (Keuleers et al., 2010). Names and lemmatized words were discarded from the word selection. Words with the same final letters or letter repetition (e.g., piep, enne) were not selected. For both high and low-frequency words, two sets of target and flanker pairs were constituted in order to manipulate the target-flanker relatedness. In the related set, target and flanker were the same word (e.g., bron bron [source source]). In the unrelated set, target and flanker were different words (e.g., bron staf [source rod]) with as few common letters and semantic relations as possible (The Appendix contains the full list of stimuli.). The set of pseudowords was created from the word set by a single letter substitution. Pseudoword targets, associated with pseudoword flankers, were used as fillers for the purpose of the lexical decision task and were not considered in the analyses.

**Figure 1:**
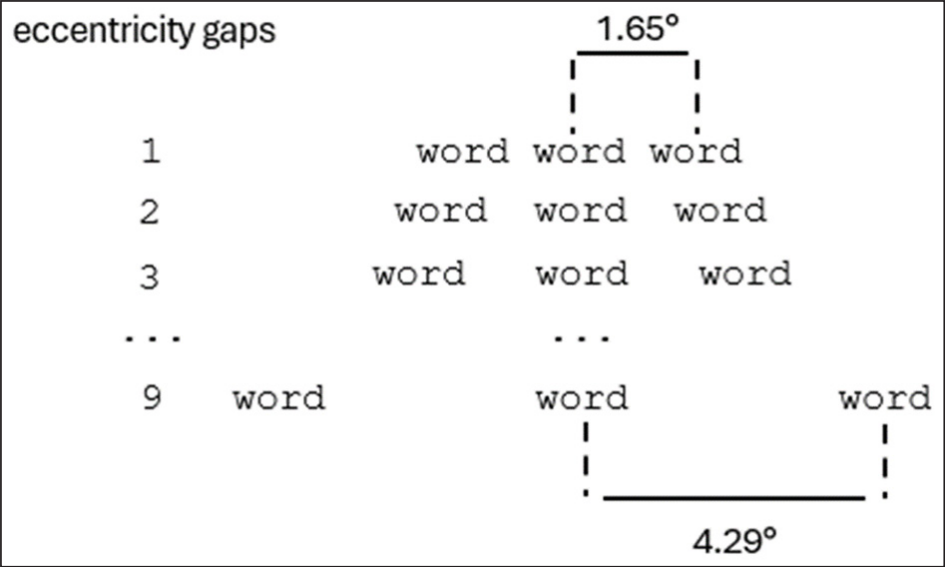
Depicting of the distribution of four-letter word stimuli (flankers and target) across nine eccentricity gaps. At the initial gap, the center of the flanker string was positioned at 1.65° of visual angle from the target string center. Subsequently, each eccentricity was located approximately one space further away from the target string center. At the final gap, the flanker string was positioned at 4.29° of visual angle from the center of the target string.

In order to track the interaction between relatedness and eccentricity, flankers could be located at different locations along a horizontal line covering 4.29° of visual angle on each side. At the first eccentricity, the center of the flanker string was located at 1.65° from the center of the screen (also the target string center), corresponding approximately to a single space separation^1^ of .33° away from the central target string, both to the left and right of it. After that, each eccentricity was one space further away from the center, up to the point where the center of the flanker string was located at 4.29° from the center of the screen. In that way there were nine possible eccentricities (see Figure 1).

The experimental design allowed us to test both eccentricity and relatedness as within-subjects factors. Items were counterbalanced across participants and conditions. To do this, we created 18 lists where all 360 items were assigned to one combination of relatedness and eccentricity.

### Procedure

**Figure 2:**
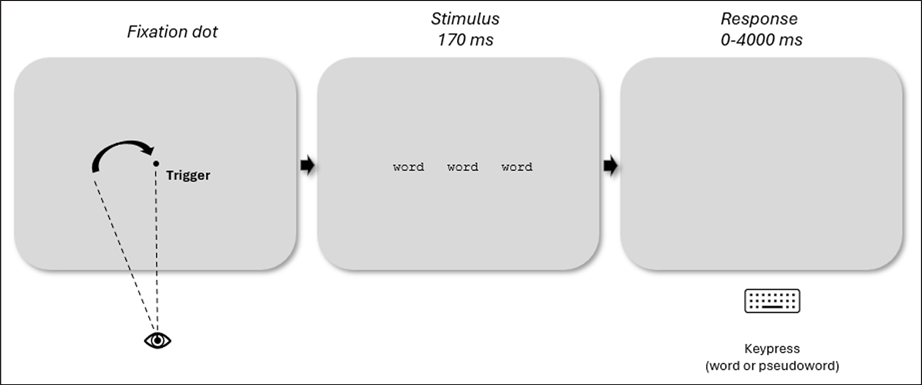
Illustration of the experimental procedure with an example of the related conditions. The central fixation dot is used as a stimulus trigger while waiting for the participant’s gaze to land. As soon as the gaze is detected, the stimulus, consisting of the target flanked by the related words located at an eccentricity of 2.64°, is displayed for a very brief period of 170 ms. The participant’s response is expected within 4000 ms and is collected via the keyboard: p for words and q for pseudowords.

An illustration of the procedure is provided in Figure 2. Participants were instructed that three strings of letters would briefly appear on the screen and that they had to quickly categorize the central string as a word or pseudoword, and only that one. After calibration of the eye tracker, the task started with a few trials in a training block which then gave way to the actual task. Each trial started with a centralized fixation dot used as trigger: When the participant’s gaze landed on the fixation dot, the target and flanking stimuli were presented for 170 ms. Participants had a maximum of 4000 ms to make their lexical decision with a right-handed (“p”) button press for word targets and left-handed (“q”) button press for pseudoword targets, using a qwerty keyboard layout. Following the response, a blank screen was presented for 1000 ms before the start of the next trial.

The eccentricity of the flankers varied from 1.65° to 4.29° of visual angle from the central target word. They could be located at 1.65°, 1.98°, 2.31°, 2.64°, 2.97°, 3.30°, 3.63°, 3.96° or 4.29° from the central target word. The 360 trials were presented in random order. A break with a duration determined by the participant was offered halfway in the experiment. The experiment started with a 20 items practice session with positive and negative feed-backs. After the practice session, the participant was notified that the experiment itself was going to start. There was no feedback during the experiment. The experiment lasted approximately 30 minutes.

### Statistical Power

Statistical power was estimated a posteriori using the simulation approach suggested by Brysbaert & Stevens (2018). We employed the Monte Carlo method using the *powerSim* function from the simR package (Version 1.0.5; Green & McLeod, 2016). The simulations yielded an estimated statistical power of 0.89 for detecting the Relatedness by Eccentricity interaction, which suggests that the study was sufficiently powered.

## Results

In order to avoid any artifact caused by low-frequency words that may have been unknown to many participants, all items with an accuracy rate under 70% were discarded. This affected 20 items in the low-frequency words set. The remaining set consisted of 160 items. Pseudoword fillers were not analyzed. Error trials (5.6%) and trials with improbable RTs below 300 ms or above 1500 ms (1.1%) were excluded. RTs were log-transformed to meet normality assumptions. A null model with a random structure including by-participant and by-item random intercepts was fitted to compute standardized residuals from the logarithmic RTs variable. All trials with standardized residuals larger than 2.5 SD*s* from the grand mean were excluded (2,5%).

The data were analyzed in R (R Core Team, 2022). Linear mixed-effects models (RTs) were fitted with the *lmer* function from lme4 (v1.1-21; Bates et al., 2014), and generalized mixed-effects models (accuracy rates) with the *glmer* function. Fixed effects were considered significant if |*t*| or |*z*| > 1.96 (Baayen, 2008). Post hoc analyses for significant two-way interactions used cell means coding and single df contrasts, implemented via the *glht* function from multcomp (v1.4-13; Hothorn et al., 2008) with normal distribution significance testing (see Table 1).

**Table 1:** Mean RTs (in milliseconds), accuracy (probabilities) for word targets (Standard deviations in parentheses) and delta (unrelated–related) are provided for each of the experimental conditions.


FLANKERS ECCENTRITY (DEGREES)	RTS/RELATEDNESS			ACCURACY/RELATEDNESS		
	
	RELATED	UNRELATED	Δ (SIGNIF.)	RELATED	UNRELATED	Δ (SIGNIF.)

1.65°	576 (74)	642 (66)	66***	.97 (.06)	.94 (.09)	–.03**

1.98°	586 (70)	624 (63)	38***	.95 (.07)	.93 (.10)	–.02 ^ns^

2.31°	580 (68)	617 (60)	37***	.95 (.09)	.93 (.09)	–.02 ^ns^

2.64°	582 (69)	613 (71)	31***	.95 (.07)	.94 (.08)	–.01 ^ns^

2.97°	586 (73)	612 (70)	26***	.95 (.08)	.93 (.09)	–.02 ^ns^

3.30°	573 (68)	601 (69)	28***	.96 (.07)	.93 (.09)	–.03 ^^^

3.63°	579 (66)	596 (67)	17 **	.96 (.07)	.95 (.08)	–.01 ^ns^

3.96°	574 (69)	598 (70)	24***	.94 (.08)	.93 (.10)	–.01 ^ns^

4.29°	574 (65)	605 (72)	31***	.96 (.08)	.94 (.09)	–.02 ^ns^


Notes. Delta (Δ) represents for the relatedness effects (unrelated–related). Accuracy rates and RTs significance values were calculated from a no-intercept model specified as following: accuracy/log10(RT) ~ 0 + relatedness:eccentricity + (relatedness|subject) + (relatedness|item). Significance levels: ***p < .001, **p < .01, *p < .05, ^^^*p* < .10 and ^ns^*p* > .10.

### Accuracy rates analyses

For the 3-way interaction model, the maximal converging random effect structure was one including by-participant and by-item random intercepts and slopes among the Relatedness by Eccentricity interaction.

In this analysis, the main effects of both Relatedness and Frequency were significant (*b* = –.90, SE = .40, *z* = –2.26; *b* = –1.37, SE = .41, *z* = –3.34; respectively) with higher accuracy in the related flanker conditions and for high-frequency words. No interaction effects were significant in this analysis.

### RTs analyses

#### Relatedness by Frequency by Eccentricity interaction

For the 3-way interaction model, the maximal converging random effect structure included by-participant and by-item random intercepts and slopes among the Relatedness by Eccentricity interaction.

There was a main effect of relatedness (*b* = .04, SE = .004, *t* = 7.86) and of word frequency (*b* = .03, SE = .006, *t* = 6.77), with faster responses in the presence of related flankers and for high-frequency words. The main effect of the eccentricity did not reach the significance (*b* = –.001, SE = .001, *t* = 1.23). The relatedness by eccentricity interaction was significant (*b* = –.003, SE = .008, *t* = –3.49). No more interactions were significant in this analysis.

To explore the effect of Relatedness at individual levels of Eccentricity, a post-hoc analysis was conducted (results are shown in Table 1). This analysis revealed a significant relatedness effect for all levels of eccentricity.

#### Polynomial analysis

The interaction between relatedness and eccentricity appears to be driven primarily by the variation in the unrelated condition, where RTs decrease with increasing eccentricity, while RTs in the related condition remain stable. To investigate this pattern further, we examined the effects of eccentricity separately for each relatedness level.

**Figure 3:**
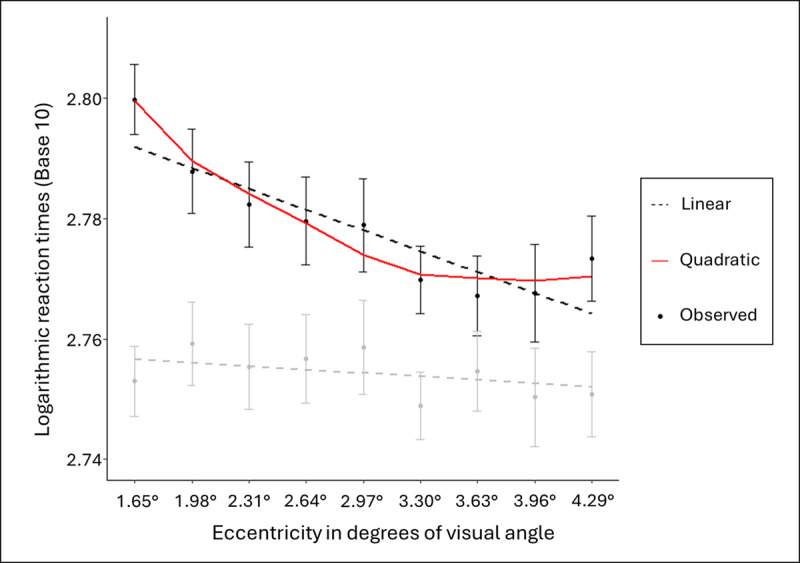
Base-10 logarithmic RTs across eccentricity in both the related (grey) and the unrelated (black) conditions. Lines represent for linear and quadratic prediction values and points for observed data. Error bars are the within-participant 95% Confidence intervals (Cousineau & O’Brian, 2014).

In the related condition, RTs showed no significant effect of eccentricity (*t* < 1, *p* > .10). In contrast, in the unrelated condition, both the linear (*b* = –.003, SE = .0005, *t* = –6.51) and quadratic (*b* = 0.23, SE = 0.07, *t* = 3.55) effects were significant, confirming a systematic decrease in RTs as eccentricity increased. A model comparison using a likelihood ratio test (*anova* function, stats package) showed no significant difference (*p* = 1) between the linear and quadratic models, preventing a clear selection of the best fit. Higher-degree polynomial models (cubic and quartic) were also tested but did not reach significance. The observed and predicted RTs across eccentricities in both related and unrelated conditions are presented in Figure 3.

## Discussion

The aim of this work was to track the integration of orthographic information within the perceptual span with a new paradigm. We tested the effects of eccentricity in the perceptual span by presenting related and unrelated flankers to the central word, placed at different locations in the parafovea. We used an eye tracking control and limited the display duration to 170 ms to ensure that participants fixated on the targets.

Our study replicated the typical pattern found in flankers task, where unrelated flankers (e.g., path rock path) lead to longer RTs compared to related flankers (e.g., rock rock rock). These relatedness effects were initially found with bigram flankers (Dare & Schillcock, 2013; Grainger et al., 2014) and later with whole-word flankers (e.g., Snell et al., 2017; Vandendaele & Grainger, 2023; Cauchi et al., 2020). Although the typical pattern was observed in the flankers task without eye movement control, the present study demonstrates that in the context of strict eye movement control – ensuring that gaze does not shift to the flankers – the same patterns emerge as in previous studies. In fact, the magnitude of the relatedness effect at the smallest eccentricity was found to exceed that typically observed in previous studies (e.g., Snell & Grainger, 2018; Cauchi et al., 2022). This could be due to the novel eye-tracking procedure that ensured stimulus onset occurred only when participants were fixating precisely at the center of the display. By constraining participants to strictly focus the center of the target word, this approach likely enhances the sensitivity of our paradigm and may amplify the relatedness effects.

Results showed that RTs in the unrelated flankers condition decreased substantially between 1.65° and 1.98°, with a less pronounced decrease along the rest of the axis, extending to 4.29°. This observation of a non-linear decrease of the unrelated flankers condition is supported by the significance of the quadratic term in the polynomial analysis, although, model comparison could not confirm that a non-linear model provides a better fit than the linear model. Despite this decrease, post-hoc analyses revealed that the relatedness effect remained significant even for the most distant flankers, suggesting that flankers were still processed at 4.29° of visual angle from the central word.

What do the results of this study tell us? They demonstrate that parallel processing extends to large distances from the fovea, reaching the outside boundaries of the parafoveal region. In the context provided by this adapted version of the flankers task, we provide further evidence that – as proposed by Grainger et al. (2014) and recently revisited by Vandendaele & Grainger (2023) – spatial integration of orthographic information occurs across multiple spatially distinct stimuli within a single processing channel for sub-lexical orthographic processing and word identification. Importantly, the present study extends this finding by showing that orthographic information at greater distances from the foveal central word still slow its recognition. This implies that unrelated flankers still exert inhibition, perhaps through lexical competition, at a large distance. This finding would be consistent with the computational model OB1-reader (Snell, van Leipsig et al., 2018; Meeter et al., 2020), which was implemented based on the theoretical framework proposed by Grainger et al. (2014). Indeed, a key feature of this model states that processing is spatially distributed, with acuity decreasing as eccentricity increases. Our results suggest that this principle holds even for very distant flankers, reinforcing the idea that spatial integration is not limited to immediately adjacent orthographic information but can extend further into the parafovea.

Importantly, our data did not support the foveal load hypothesis (Henderson & Ferreira, 1990), which predicts that less attention would be allocated to parafoveal flankers when the foveal target word is a low frequency item. A reduction in parafoveal processing due to foveal load was previously reported by Vignali et al. (2019), who found that flanker processing decreased when the central target was a pseudoword. A similar effect was reported for semantic information: when a foveal word is semantically unexpected or incongruent, parafoveal preview benefits decrease (Payne et al., 2016). This suggests that foveal processing difficulty modulates the extent to which parafoveal words are integrated. However, our study did not reveal any difference in parafoveal processing as a function of lexical frequency: while low-frequency words led to longer RTs than high-frequency words, no interaction was observed between lexical frequency and other experimental factors. This could be explained by the fact that the experimental manipulation employed in these brief presentations primarily engages early orthographic processing mechanisms, *i.e.*, low-level processes which are not sensitive to lexical frequency. Consequently, lexical frequency influenced the foveal target recognition but does not appear to have directly modulate spatial integration in parafovea.

Finally, we highlight that this paradigm, as a laboratory task, does not fully reflect everyday reading. Indeed, in a natural reading context, attention may be diluted by the many words simultaneously visible. The present study builds on the work of Fournet et al. (2023), where we demonstrated that proximal bigram flankers interfere more with target word processing than distant flankers. However, in Fournet et al. we did not quantify these effects in relation to visual eccentricity. The present work is an initial attempt at quantifying the spatial integration of orthographic information within the perceptual span using the change in location of the flanker relatedness manipulation.

Our results are consistent with the findings of McConkie & Rayner (1975) regarding the perceptual span. Specifically, the higher eccentricity tested in this study, 4.29°, corresponds to a spacing of 14–15 characters between the center and the last letter of the flanker. By using this new paradigm, different from the moving window technique, we provide an alternative way of assessing how orthographic information is integrated within the perceptual span.

## Conclusion

This study introduced a novel version of the flankers task to examine how orthographic information is integrated across the perceptual span. Our results showed that orthographic information is not processed uniformly across the perceptual span, and we proposed a metric to describe this decline. We suggest that future studies should focus on developing readers to track the developmental progression of orthographic processing within the perceptual span using this paradigm. This study examined the influence of parafoveal information with bilateral rather than unidirectional flankers, given the attentional asymmetry towards the right with Latin alphabets (see Ducrot & Grainger, 2007 for a review), it would be valuable to explore how the central word processing is impacted by the flanker location, whether on the right or left. This could be addressed using unilateral flanker displays, as in Snell and Grainger (2018), or through the presentation of lateralized non-lexical characters (*e.g.*, “#”), as in Cauchi et al. (2024). Furthermore, recent findings from Lázaro et al. (2025) suggest that the contribution of flankers may depend not only on their spatial location but also on the degree of orthographic overlap with the target, emphasizing the need to disentangle spatial and linguistic influences on parafoveal processing. Finally, as underlined by Veldre et al. (2023b), the relationship between eccentricity and orthographic processing is not well defined in current reading models, and we agree that it would be interesting to see whether they could account for these findings.

## Data Accessibility Statement

Data, statistical analysis files and materials are available from this OSF link: https://osf.io/2eu7s/?view_only=aa13bc0fb38648b396bcbdec4d188146.

## Footnotes

To account for cortical magnification in the peripheral region, each level of eccentricity was calculated to precisely correspond to the visual angle degree assigned to it. Therefore, center-to-center eccentricities were converted from degrees into pixels instead of using fixed typographic spaces. This results in a linear increase in visual degrees of 0.33° at each eccentricity level but a slight a non-linearity in the spacing in pixels on the screen.

## Appendix

**Table:** List of target words and unrelated flankers (set of high-frequency words).

TARGET WORD	UNRELATED FLANKER	TARGET WORD	UNRELATED FLANKER	TARGET WORD	UNRELATED FLANKER

adem	rots	heks	blad	orde	zeep

alle	truc	hond	maal	park	geil

auto	loon	hoog	veld	pers	bouw

berg	klop	huis	pech	plek	grot

boot	live	hulp	vlag	plus	knie

bord	muis	jong	sire	pond	blut

bril	munt	jouw	tank	punt	nood

bron	staf	jury	roos	ramp	mode

buik	nest	kamp	west	reis	doei

club	verf	kast	trui	ring	knal

code	lamp	keer	zand	slag	ruil

dame	knop	kind	vers	slot	aard

deur	soep	kist	borg	snap	golf

dief	blok	klap	gids	stad	beet

dier	ofzo	klok	buit	stof	ezel

doen	wijf	laag	poen	teef	luxe

echt	lied	leuk	rand	tent	pols

eeuw	zoet	lijf	klem	tijd	wolf

enig	riem	lijn	zout	toen	geur

erom	link	meid	tape	tong	hemd

eten	plat	melk	zone	tuin	stam

fijn	halt	mijn	kaas	vlug	wond

flat	leed	moed	pijp	voet	stok

fles	bond	naam	roze	voor	stal

foto	daad	neef	coma	vuil	gene

gast	boer	neus	tuig	werk	vies

gave	fris	niet	lord	wild	duim

geen	hout	noch	zuur	zelf	joch

glas	wiet	olie	helm	zoon	gras

goud	fase	open	doof	zwak	grof

**Table:** List of target words and unrelated flankers (set of low-frequency words).

TARGET WORD	UNRELATED FLANKER	TARGET WORD	UNRELATED FLANKER	TARGET WORD	UNRELATED FLANKER

baat	lier	kruk	biep	puik	tred

balk	spil	kuis	zoem	rund	nipt

beuk	mint	kurk	zeef	slak	trog

bieb	klip	kwal	pees	sluw	deck

bijl	tuba	laan	oker	smak	berm

boks	wurm	lade	kolf	sneu	drup

boog	mals	loer	wrat	snor	edel

buur	knol	lomp	waas	snot	mime

darm	enen	luid	hoes	spin	kaap

doek	plug	mank	teug	spit	grom

doop	reuk	mast	bres	stek	hoon

duif	pens	meel	vilt	stip	orka

dump	kier	mest	laks	stro	kiwi

galg	hiel	mild	brul	stug	jive

gans	vete	milt	zool	vaat	tros

gips	kaft	moes	lauw	vonk	aula

hark	föhn	muil	knor	vouw	knel

heup	foei	naad	loof	waak	leus

jeuk	riet	norm	gesp	waan	gnoe

kalf	bout	ober	fors	werf	judo

klik	dauw	oven	zeug	zalf	bonk

knik	hars	peil	hork	zoen	gril

knus	welp	poot	hels		

kras	merg	prop	kiem		

*-The target words that achieved an accuracy rate of less than 70% were the following: alom, ambt, cast, clou, doch, doeg, dunk, fern, fuif, hens, huls, loge, nors, pact, pief, poel, ruis, sein, smid, vrek.
